# Outcomes of ECMO support with polypropylene membrane during pandemic times: a retrospective cohort study

**DOI:** 10.1186/s12890-023-02753-5

**Published:** 2024-01-19

**Authors:** Natalia Valenzuela-Faccini, Claudia Poveda-Henao, Catalina Flórez-Navas, Michel Pérez-Garzón, Natalia Boada-Becerra, Mario Mercado-Diaz, Patricia Salcedo, Henry Robayo-Amortegui

**Affiliations:** 1 Critical care resident, Universidad de La Sabana, Chía, Colombia; 2 Critical Medicine and Intensive Care, Intensive care department Fundación Clínica Shaio, Bogotá DC, Colombia; 3 ECMO group Fundación Clínica Shaio Perfusionist intensive care department, Fundación Clínica Shaio, Bogotá DC, Colombia

**Keywords:** ECMO, SARS-CoV-2, Polypropylene membranes, Poly-methylpentene membranes, Complications

## Abstract

**Background:**

The SARS-CoV-2 pandemic resulted in shortages of supplies, which limited the use of extracorporeal membrane oxygenation (ECMO) support. As a contingency strategy, polypropylene (PP) oxygenation membranes were used. This study describes the clinical outcomes in patients on ECMO with PP compared to poly-methylpentene (PMP) oxygenation membranes.

**Methods:**

Retrospective cohort of patients in ECMO support admitted between 2020 and 2021.

**Results:**

A total of 152 patients with ECMO support were included, 71.05% were men with an average age of 42 (SD 9.91) years. Veno-venous configuration was performed in 75.6% of cases. The PP oxygenation membranes required more changes 22 (63.1%), than the PMP Sorin® 24 (32,8%) and Euroset® 15 (31,9%) (p.0.022). The main indication for membrane change was low oxygen transfer for PP at 56.2%, Sorin® at 50%, and Euroset® at 14.8%. Renal replacement therapy was the most frequent complication with PP membrane in 22 patients (68.7%) Sorin® 25 patients (34.2%), and Euroset® 15 patients (31.9%) (p 0.001) without statistically significant differences in mortality.

**Conclusion:**

PP oxygenation membranes was a useful and feasible strategy. It allowed a greater disponibility of ECMO support for critically ill in a situation of great adversity during the SARS-CoV-2 pandemic.

## Background

The severe acute respiratory syndrome coronavirus 2 (SARS CoV-2) pandemic caused an unprecedented overload on healthcare systems with a shortage of medical resources for patient care [1]. The use of extracorporeal membrane oxygenation (ECMO) support increased exponentially due to the rise in severe cases of acute respiratory distress syndrome (ARDS) and refractory hypoxemia [2, 3], in addition to patients with other conditions that required this support [4, 5].

A vital component of the ECMO circuit is the oxygenation membrane that allows the adequate gas exchange [6]. In the 1980s, polymeric materials were developed, including polypropylene (PP) oxygenation membranes, with micropores that allow high gas transfer, low priming volumes, and low resistance. Nowadays, these membranes are limited to cardiac surgery with a recommended usage of no more than eight hours due to the risk of plasma leakage [7]. Currently, polymethylpentene (PMP) is the material of choice for ECMO oxygenators, providing safer support with a non-porous membrane that separates blood from the gas phase, reducing hemolysis and plasma leakage, and with a lower systemic inflammatory response and thrombosis risk, allowing for a longer lifespan [8, 9].

The increase of cases of severe ARDS during the SARS-CoV-2 pandemic exceeded the capacity of ECMO centers, with a shortage of supplies, including PMP oxygenation membranes [10]. Therefore, using PP oxygenators was considered a contingency measure for extracorporeal support during the health emergency. This study aims to describe the clinical outcomes in patients with ECMO support that required PP oxygenation membranes compared to PMP membranes during the SARS-CoV-2 pandemic.

## Materials and methods

A retrospective cohort study was conducted in patients admitted to the intensive care unit (ICU) and cannulated for ECMO support at Fundación Clínica Shaio in Bogotá, Colombia, a reference center for ECMO support and a member of ELSO, during the SARS-CoV-2 pandemic from January 1, 2020, to December 31, 2021. Data were collected retrospectively using the REDCAP® tool with institutional ethical and research committee approval; informed consent for demographic, physiological, and hospital-outcome data analyses was not obtained because this observational study did not modify existing diagnostic or therapeutic strategies [11].

### Population and study

**Fig. 1:**
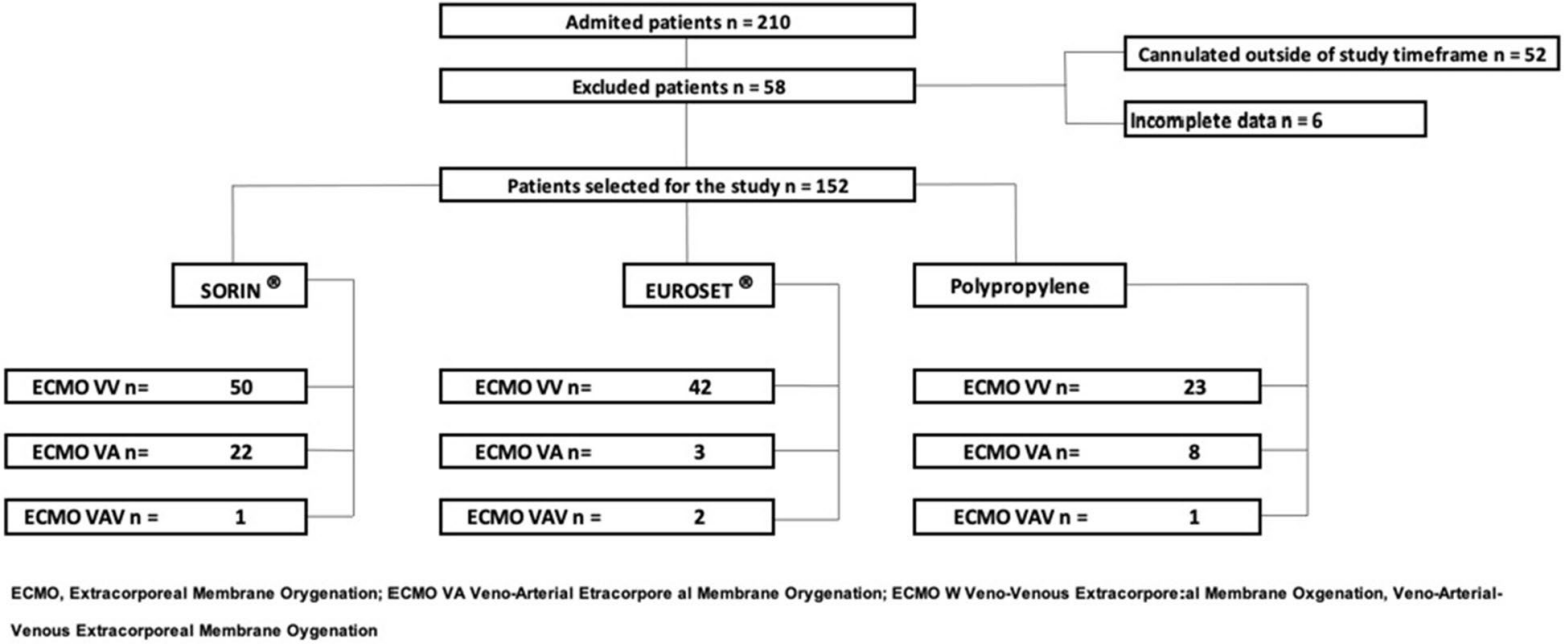
Flow chart of subject enrollment in the study

Patients over 18 years of age with acute respiratory failure and/or refractory cardiogenic shock requiring veno-venous (VV), veno-arterial (VA), or venoarterial-venous (VAV) ECMO were included, according to the criteria established by ELSO [12]. Due to the shortage of PMP oxygenation membranes and previous experience with satisfactory results in cardiac surgery, the PP oxygenation membranes were used. This decision was discussed and approved by the ECMO medical board and the institutional ethics committee after carefully evaluating the benefits and risks associated with their use [13]. Thus, prioritization was based on ICU occupancy during the pandemic, and allocation of oxygenation membranes was based on availability at the time of cannulation [14]. Available PMP membranes were Sorin® and Euroset®, and PP membranes were Inspire 8F®. Based on anthropometric data and the need for greater contact surface area to achieve oxygenation goals in some patients, depending on availability, a second oxygenation membrane was placed in parallel, either PP or Eurosets® PMP. Patients who died within the first 6 hours, were cannulated outside the study period, previously supported with ECMO, had incomplete data, or unclear complications or cause of death were excluded (Fig. 1).

For patients who required air transport, only PMP membranes were used because there was no information on the behavior of PP membranes at high altitudes. The decision to change oxygenation membranes was based on a decrease in gas transfer according to the manufacturer’s recommendations for each membrane [12], plasma leakage was defined as an increase in polypropylene micropore permeability with evidence of water loss and bubbles through the oxygenator, and hemolysis as the presence of anemia, an increase in indirect bilirubin, and lactate dehydrogenase [12, 15]. Mortality data were extracted from death records and reported in electronic medical records.

Sociodemographic characteristics, comorbidities, clinical variables at admission, and previous paraclinical findings before ECMO support were studied, and complications during support were summarized. The Sequential Organ Failure Assessment (SOFA) [16], Acute Physiology and Chronic Health Evaluation (APACHE II) [17], Respiratory ECMO Survival Prediction (RESP) score [18], and Survival after Veno-Arterial ECMO (SAVE) score [19] were calculated according to their original studies within the first 24 hours of admission. The indication for membrane change was evaluated, and finally, outcomes related to complications, days of mechanical ventilation, ICU stay, hospital stay, and mortality were analyzed.

### Statistical analysis

Frequencies and percentages were presented as qualitative variables, and quantitative variables were presented as medians and interquartile ranges if their distribution was normal and as means and standard deviations if their distribution did not meet the parameters of normality evaluated by the Shapiro-Wilk test. Trends over time were analyzed using Spearman’s rank correlation test for ordinal variables and Wilcoxon’s rank-sum test for dichotomous variables. A *p*-value less than 0.05 was considered statistically significant. STATA was used for the statistical analysis. (Version 17.0, Stata corp. College Station, Texas, USA) with a license for it.

## Results

Of the 152 patients selected for the study, 108 (71%) were male with an average age of 42 (SD 9.91) years, and a body mass index (BMI) of 29.7 (SD 5.15). The relevant pathological history included arterial hypertension (18.4%), diabetes mellitus (14.4%), and smoking (4.6%). At the time of cannulation, the patients had a SOFA Score of 7 points (IQR 5–9), an APACHE II Score of 9 points (IQR 5.5–14), a RESP Score of 3 points [2–4] in the case of respiratory ECMO, and a SAVE Score of 0 points (− 3 to 0) in the case of cardiac ECMO. The predominant etiology was SARS-CoV-2 infection in 117 patients (76.9%), which is why VV-ECMO was the most commonly used configuration in 115 patients (75.6%), followed by VA-ECMO in 33 patients (21.7%), VAV configuration was used in 4 cases (2,7%). The use of PMP oxygenation membranes were Sorin® 73 (48%), Euroset® 47 (30.9%), while Inspire® PP-type membranes were 32 (21%). PP oxygenation membranes required 22 (63.1%) changes compared to Sorin® PMP membranes with 24 (32.8%) and Euroset® with 15 (31.9%) (p 0.022) changes, and the main indication for membrane change was low oxygen transfer for PP in 56.2%, Sorin® 50%, and Euroset® 14.8% (Table 1).

**Table 1:** Baseline characteristics

Characteristics	All Patients *n* = 152	Dead = 58	Alive = 94	*P* value
Age in years, median (de)	42 (9.91)	45.5 (9.44)	40 (9.97)	0.007*
Male, n (%)	108 (71.05%)	45 (77.59%)	63 (67.02%)	0.163
Weight Kg, median (de)	85.5 (17.08	86.1 (16.59)	85.2 (17.47)	0.761
Body mass index, kg/cm2 median (de)	29.7 (5.15)	29.8 (5.22)	29.6 (5.13)	0.817
Days in ICU - M (RIC)	24.1 (13.4–39.9)	14.4 (6.8–26.7)	30.5 (18.8–48.2)	< 0,001*
Days in mechanical ventilation	17.4 (10.5–26.7)	15.4 (8.77–23.3)	19 (12.9–27.2)	0.669
Days hospitalized	29(16–51)	17 (8–29)	39 (25–57)	0,005*
**Comorbilities, n (%)**				
Arterial hypertension	28 (18.4%)	13 (22.4%)	15(15.9%)	0.319
Diabetes	22 (14.4%)	7 (12.1%)	15 (15.9%)	0.508
Dislypidemia	6 (3.9%)	2 (3.4%)	4 (4.2%)	0.804
Asthma	8 (5.2%)	4(6.9%)	4 (4.2%)	0.479
Active smoker	7 (4.6%)	3 (5.1%)	4 (2%)	0.793
**Laboratories previous to cannulation, median (ds)**				
Potassium meq/l	4.01 (0.96)	3.78 (1.27)	4.15(0.66)	0.044*
Creatinine mg/dl	1.60(3.82)	1.54 (1.16)	1.64 (4.78)	0.847
Ureic nitrogen	27.8 (18.9)	30.8 (19.61)	26 (18.3)	0.134
Infection, n (%)				
SARS-CoV-2	117(76.9%)	48(82.7)	69(73.4%)	0.183
**Mode of Support (%)**				0.881
Venovenous ECMO	115(75.6%)	44 (75.8%)	71 (75.5%)	
Venoarterial ECMO	33 (21.7%)	13 (22.4%)	20 (21.2%)	
Venoarteriovenous ECMO	4 (2.7%)	1 (1.72%)	3 (3.19%)	
**Membranes, n (%)**				0.118
Sorin	73(48%)	22(37.9%)	51(54.2%)	
Euroset	47(30.9%)	20(34.4%)	27(28.7%)	
Polypropilane-Inspire	32 (21%)	16(27,5%)	16(17%)	
**Severity score M (RIC)**				
SOFA Score	7(5–9)	8(6–11)	7(4–9)	0,101
APACHE II Score	9(5.5–14)	11 (6–17)	8 (5–12)	< 0,001*
Oxygen debt DEOx	“- 1.16((−13.2) -10.8)	5.01 ((−9.8)-12.8)	“-4.44((−15.2)-6.67)	< 0,001*
RESP	3(2–4)	2(1–4)	4(2–5)	0.255
SAVE	0(−3 a 0)	0 (−2 a 1)	“-5 (−3–0)	0.494
**Complications, n (%)**				
Mechanical	34 (22.3%)	13(22.4%)	21(22.3%)	0.647
Mayor bleeding	20 (13.1%)	10(17.2%)	10(10.6%)	0.242
Neurological	27(17.7%)	20(34.4%)	7(7.4%)	< 0,001*
Cardiovascular	18(11.8%)	11(18.9%)	7(7.4%)	0.033*
Renal	39(25.6%)	21(36.2%)	18(19.1%)	0.019*
Infection	115(75%)	29(67.7%)	76(80.8%)	0.05*
Oxigenator failure	22(14.4%)	8(13.7%)	14(14.8%)	0.85
Delirium	83(54.6%)	13(2.4%)	70(74.4%)	< 0,001*
Renal replacement therapy	62(40.7%)	24(41.3%)	38(40.4%)	0.97

SE, estándar deviation, M median, RIC interquartile range, ECMO extracorporeal mechanical oxygenation, SOFA, Sequential organ failure assessment; APACHE, acute physiology and chronic health disease classification system;*p < 0,05

The main complication was delirium in 39 (53.4%), 28 (59.5%), and 16 (50%) (p 0.676) for patients in the Sorin®, Euroset®, and PP membrane groups, respectively, followed by acute kidney injury requiring replacement therapy (RRT) in the PP group in 22 patients (68.7%), Sorin® 25 patients (34.2%), and Euroset® 15 patients (31.9%) (p 0.001). Major bleeding occurred in patients with Sorin® membranes (10.9%), Euroset® membranes (12.7%), and PP membranes (18.7%) (p 0.551), while oxygenator failure occurred in Sorin® 12.3%, Euroset® 14.8%, and PP 18.7%, mainly due to plasma leakage (p 0.687) (Table 2).

**Table 2:** Membrane complications

Characteristics	Sorin =73	Euroset =47	Polypropilene =32	P Value
Masculine	46 (63%)	38 (80,8%)	24 (75%)	0.094
Number of membrane changes	24 (32.8%)	15 (31.9%)	22(63.1%)	0.022*
Mechanical ventilation days	16.1 (11.3–22.6)	17.2 (10.6–27)	17.8 (10.5–28.7)	0.37
ICU stay	22 (8.8–44)	25.6 (14.2–45)	22.9 (15.3–38.5)	0.4
Hospital stay	25 ((14–58)	30.5 (18–52.5)	27 (16–46)	0.378
Mortality	22 (30.1%)	20 (42.5%)	16 (50%)	0.118
Membrane change indication n (%)				0.001*
Low transference	12(50%)	7(14.8%)	18(56.2%)	
Hemolysis			2 (4.2%)	
Infection	3(4.1%)	1(2.1%)		
Thrombosis	2(2.7%)			
**Complications n (%)**				
Mayor bleed	8(10.9%)	6 (12.7%)	6 (18.7%)	0.551
Renal replacement therapy	25 (34.2%)	15 (31.9%)	22 (68.7%)	0.001*
Bomb failure	2(2.7%)	1(2.1)		0.647
Oxigenator failure	9(12.3%)	7 (14.8%)	6(18.7%)	0.687
Delirium	39 (53,4%)	28 (59,5%)	16 (50%)	0.676
**Support mode, n(%)**				0.063
Venovenous ECMO	50 (68,4%)	42 (89,3%)	23 (71,8%)	
Venoarterial Venoarterial	22 (30,1%)	3 (6,38%)	8 (25%)	
Venoarterial venous ECMO	1 (1,3%)	2 (4,2%)	1(3,1%)	
**Type of infection, n (%)**				
SARS-CoV-2	51 (69.8%)	41(87.2%)	25 (78.1%)	0.086

ICU intensive care unit ECMO,extracorporeal oxigenation membrane;*p < 0,05

The mechanical ventilation days were 16.1 (IQR 11.3–22.6) days for Sorin®, 17.2 (IQR 6–27) for Eurosets®, and 18.8 (IQR 10.5–28.7) with PP (p 0.37). ICU stay was 22 (IQR 8.8–44) days for Sorin®, 25.6 (IQR 14.2–45) for Euroset®, and 22.9 (IQR 15.3–38.5) for Inspire® (p 0.4). Mortality with PP membranes was 16 (50%) patients, Sorin® 22 (30.1%), and Euroset® 20 (42.5%) (p 0.118).

## Discusion

Our study describes the outcomes of patients on ECMO support during the SARS-CoV-2 pandemic with PP and PMP oxygenation membranes. There was a higher number of PP membrane changes due to low oxygen transfer, with an increased need for renal replacement therapy (RRT), without statistically significant differences in ICU stay, hospital stay, days of mechanical ventilation, and mortality.

Patients who received PP oxygenation membranes showed a higher change rate of 63.1% (p 0.022) due to low oxygen transfer of 56.2% (p 0.001). This is attributed to the pore characteristics of PP membranes, which generate a hydrophilic surface that allows greater contact with blood cell phospholipids and leads to plasma leakage, limiting the membrane’s lifespan to a few hours compared to PMP membranes, which are more hydrophobic and have greater durability in ECMO therapy [20, 21]. An ELSO report of 1035 SARS-CoV2 patients supported by ECMO found that only 8% of patients required a membrane change [22]. In a retrospective cohort in Chile of patients with SARS CoV-2 ARDS, an oxygenator failure rate of 23.5% was described [23]. Comparing these results, it was found that the percentage of membrane changes in our study was higher. This could be explained, first by the exclusive use of PMP membranes in these studies, second by the limitation of membrane size at the time of cannulation, as anthropometric data showed a need for greater contact surface area to achieve oxygenation goals, which required the use of a double membrane. Finally, the geographical location of Bogotá, which is at an altitude of 2600 m above sea level, changes oxygen pressures and may be a factor that affects oxygen transfer of oxygenators tested at 1200 m above sea level [24, 25].

Despite the improved survival of patients on ECMO, adverse effects are common, such as acute kidney injury (AKI), infection, thrombosis, and hemorrhage [26]. In our study one of the main complication identified was the need of renal replacement therapy (RRT) which was higher with the PP oxygenation membrane group (68.7%) compared to Sorin® PMP (34.2%) and Eurosets® (31.9%) (p 0.001). As for the factors that were identified with a higher need of renal replacement was acute kidney injury prior to cannulation and the SARS COV 2 infection in 76.9% of the patients, this has been associated with more AKI and need of RRT in prior studies [27]. Overall, available evidence suggests that acute kidney injury is a frequent and significant complication in patients undergoing ECMO support with an incidence ranging from 30 to 60% [28]. Physiological factors associated with this condition can be explained by hypoperfusion, inflammation, and exposure to foreign surfaces such as oxygenators, in addition to preexisting renal injury that worsens this condition [29]. A systematic review by Mitra et.al reports that VA-ECMO requires more renal replacement therapy 72% with a mortality rate of 63% [30]. In a retrospective analysis conducted by Haneya A. and his team with 289 patients who received ECMO therapy for severe acute respiratory failure, 193 patients developed acute kidney injury, the primary outcome of this study was a significantly higher mortality rate (62% vs. 33%) compared to patients who did not develop kidney injury [31]. Additionally, a meta-analysis conducted by Cheng et al. indicates that patients with VA ECMO for cardiogenic shock have acute kidney injury in 55% of cases, with a need for renal replacement therapy in 40% of cases [32]. On the other hand, in patients with VV ECMO for SARS-CoV-2, the need for renal therapy was 46% [33].

Mortality in patients with severe ARDS is high, and the ECMO strategy in appropriately selected patients may lead to a reduction in the 90 day mortality described by Combes et al. [33]. In a meta-analysis of patients on ECMO for SARS-CoV-2, a mortality rate of 37.1% was reported compared to 90% in cases with severe ARDS without access to this support [10, 34]. In reported studies from Germany and Israel, ECMO mortality during the pandemic ranged from 50 to 70%, associated with a lack of resources and little experience in the centers studied [35, 36]. Ling et al. conducted a meta-analysis of 52 studies comprising 18,211 patients to characterize changes in mortality over time with a pooled mortality 48.8%. Of which the factors associated with higher mortality were age, the use of corticosteroids and the prolonged duration of extracorporeal therapy. When evaluating mortality according to the oxygenation membranes used we found an overall mortality rate of 38.1%, with no differences between PP and PMP membranes (p 0.118). The outcomes in terms of days of mechanical ventilation, ICU, and hospital stay were similar. Extracorporeal membrane oxygenation (ECMO) has been used extensively for acute respiratory distress syndrome (ARDS).

We acknowledge several limitations in our study. First, being a retrospective study there is a risk of information bias since it depends on the quality of the records; however, the information program used by the institution has a vast collection of variables concurrently gathered by medical personnel trained by the research group. The selection of the use of PMP vs PP membrane was based upon the availability of the membranes during the pandemic thus there was no randomization and could be a bias when analyzing the outcomes. We used various statistical methods to reduce biases and confounding effects, which prevent causal conclusions. Also, being a single-center study generates heterogeneity in clinical practice patterns; especially, there was no protocolized randomization for the use of membranes this was done based on the availability at the time. We acknowledge that in our observational study there is lack of robust risk adjustment by absence of an adjusted analysis with a propensity score and could increase the confounders. Future research is proposed with propensity analysis to determine these confounding factors.

## Conclusion

PP oxygenation membranes are used as a backup option in critical situations, particularly in contingency cases, with similar outcomes to PMP membranes in critically ill patients. Some findings suggest that the use of PP membranes may be associated with a higher incidence of renal injury, although this could have a multifactorial cause and patients may be more severely ill in comparison. Ultimately, the use of PP membranes may be beneficial in ECMO patients without significant complications or mortality.

## Data Availability

The datasets used and/or analyzed during the current study are available from the corresponding author on reasonable request.

## Abbreviations

(ELSO®): Extracorporeal Life Support Organization
(ECMO): Extracorporeal Membrane Oxygenation
(ECMO VA): Veno-Arterial Extracorporeal Membrane Oxygenation
(ECMO VV): Veno-Venous Extracorporeal Membrane Oxygenation
(ECMO VAV): Veno- Arterial- Venous Extracorporeal Membrane Oxygenation
(ARDS): Acute Respiratory Distress Syndrome
(SARS-CoV-2): Severe Acute Respiratory Syndrome Coronavirus 2
(PMP): Polymethylpentene
(PP): Polypropylene
(ICU): Intensive Care Unit
(SOFA): Sequential Organ Failure Assessment
(APACHE II): Acute Physiology and Chronic Health Evaluation II
(SAVE): Survival after Veno-Arterial ECMO
(RESP): Respiratory Extracorporeal Membrane Oxygenation Survival Prediction
(DEOx): Oxygen debt
(USA): United States of America
(STATA): Statistical Analysis of Data
(SD): Standard Deviations
(Stroke): Stroke
(COVID-19): Coronavirus Disease 2019
(BMI): Body Mass Index
